# Structural Mapping of Mutations in Spike, RdRp and Orf3a Genes of SARS-CoV-2 in Influenza Like Illness (ILI) Patients

**DOI:** 10.3390/v13010136

**Published:** 2021-01-19

**Authors:** Bandar Alosaimi, Asif Naeem, Majed F. Alghoribi, Lilian Okdah, Maaweya E. Hamed, Ahmad S. AlYami, Athari Alotaibi, Mushira Enani

**Affiliations:** 1 Department of Research labs, Research Center, King Fahad Medical City, Riyadh 11525, Saudi Arabia; anaeem@kfmc.med.sa; 2 College of Medicine, King Fahad Medical City, Riyadh 11525, Saudi Arabia; 3 King Abdullah International Medical Research Center, Riyadh 11211, Saudi Arabia; alghoribima@ngha.med.sa (M.F.A.); okdahli@ngha.med.sa (L.O.); 4 College of Science, King Saud University, Riyadh 11451, Saudi Arabia; Mawdalla@ksu.edu.sa; 5 Pathology and Clinical Laboratory Medicine Administration, King Fahad Medical City, Riyadh 11525, Saudi Arabia; asalyami@kfmc.med.sa; 6 General Administration for Research and Studies, Ministry of Health, Riyadh 11176, Saudi Arabia; otaibi-af@moh.gov.sa; 7 Medical Specialties Department, King Fahad Medical City, Riyadh 11525, Saudi Arabia; menani@kfmc.med.sa

**Keywords:** silent transmission, NGS, influenza like illness, COVID-19, influenza vaccine

## Abstract

In December 2019, the emergence of SARS-CoV-2 virus in China led to a pandemic. Since both Influenza Like Illness (ILI) and COVID-19 case definitions overlap, we re-investigated the ILI cases using PCR for the presence of SARS-CoV-2 in 739 nasopharyngeal swabs collected from November 2019 to March 2020. SARS-CoV-2 RNA was found in 37 samples (5%) collected mostly during February 2020. It was followed by confirmation of evolutionary and spatial relationships using next generation sequencing (NGS). We observed that the overall incidence of ILI cases during 2019–2020 influenza season was considerably higher than previous years and was gradually replaced with SARS-CoV-2, which indicated a silent transmission among ambulatory patients. Sequencing of representative isolates confirmed independent introductions and silent transmission earlier than previously thought. Evolutionary and spatial analyses revealed clustering in the GH clade, characterized by three amino acid substitutions in spike gene (D614G), RdRp (P323L) and NS3 (Q57H). P323L causes conformational change near nsp8 binding site that might affect virus replication and transcription. In conclusion, assessment of the community transmission among patients with mild COVID-19 illness, particularly those without epidemiological link for acquiring the virus, is of utmost importance to guide policy makers to optimize public health interventions. The detection of SARS-CoV-2 in ILI cases shows the importance of ILI surveillance systems and warrants its further strengthening to mitigate the ongoing transmission of SARS-CoV-2. The effect of NS3 substitutions on oligomerization or membrane channel function (intra- and extracellular) needs functional validation.

## 1. Introduction

The novel Severe Acute Respiratory Syndrome Coronavirus 2 (SARS-CoV-2) emerged in Wuhan, China, on 31 December 2019, after several reported cases of pneumonia initially [1]. On 30 January 2020, the World Health Organization considered the SARS-CoV-2 outbreak as a public health emergency of international concern [2]. After more countries started to experience clusters of SARS-CoV-2 cases with community transmission, the WHO on 12 March 2020 declared the COVID-19 outbreak as a pandemic [2]. By December 21, 2020, the SARS-CoV-2 spread to more than 200 countries with 76,934,266 confirmed cases and 1,695,386 deaths globally [3]. Clinical presentations of COVID-19 range from no symptoms (asymptomatic) to severe disease, which can lead to death [4]. While more than 80% of COVID-19 cases are mild or moderate respiratory infections, without pneumonia manifestations, dyspnea and hypoxia, hospitalization due to severe (15%) and critical disease (5%) carries major morbidity and mortality [5].

Transmission of COVID-19 occurs via respiratory droplets and close physical contact. Aerosol transmission could be possible in a hospital setting during bronchoscopy, endotracheal intubation, cardiopulmonary resuscitation and any procedures that generate aerosols [6,7]. The incubation period of SARS-CoV-2 was estimated to be 2–14 days, but it is generally expected to be around 5 days, and 12-13 days as the period from infection to symptoms onset [8]. This data also revealed that the doubling time of COVID-19 estimated to be about 7 days, while the basic reproduction number (R naught) on average is 2–4. The undocumented but infectious cases are critical epidemiological factors that play a key role in the pandemic potential of an emergent respiratory tract viral infection [9,10]. These undocumented SARS-CoV-2 cases can have mild self-limited, or no symptoms and hence go undocumented or misdiagnosed, and facilitate the rapid transmission of SARS-CoV-2 [11,12]. The undocumented COVID-19 cases in China had resulted in the transmission of SARS-CoV-2 to a large number of people [8]. Transmission of SARS-CoV-2 from undocumented cases (mild or no symptoms) estimated to be about 86.2% COVID-19 cases worldwide [11,12]. Moreover, the transmission rate of undocumented SARS-CoV-2 infections was estimated to be 55% of documented COVID-19 cases [11,13,14]. Globally, it was estimated that about two-thirds of COVID-19 cases exported from China have remained undocumented [11,13,14]. Asymptomatic COVID-19 cases seem to account for approximately 40% to 45% of SARS-CoV-2 infections. Remarkably, the asymptomatic and symptomatic COVID-19 cases have the same amount of viral load and viral shedding; this data suggests a similar potential for SARS-CoV-2 transmission [15,16]. Currently, there is a piece of accumulating evidence indicating that a large number of SARS-CoV-2 confirmed cases are infected via asymptomatic cases [15,16,17]. Remarkably, too, only 3%–10% of the asymptomatic COVID-19 cases can become symptomatic [18].

Respiratory tract infection (RTI) comprises a major complicating factor that affects the health of individuals and the economy of communities. The profound medical impact of the Influenza Like Illness (ILI) brings it as one of the leading causes of distress, absenteeism and hospitalization worldwide [19]. Identifying the etiologic agent for cases of ILI is difficult due to the implication of a number of microorganisms either alone or in a combination in the clinical presentation of the ILI [20]; thus, the etiologic agent of ILI cannot be identified only on the basis of clinical presentation and symptoms. According to the WHO, ILI is defined as the presence of fever greater than or equal to 38°C, in addition to sore throat or cough [21]. After 1–4 days of illness, several respiratory signs and symptoms such as headache, nausea and malaise appear. A recent study revealed that mild and/or moderate COVID-19 patients have typical influenza-like symptoms, including fever more than 38 °C, cough or sore throat [22]. Moreover, the epidemiological characteristics of COVID-19 cases showed a clear overlap between symptoms and clinical characteristics of the mild and moderate COVID-19 cases and ILI definition [23].

Several vaccine development platforms (recombinant protein vaccines based on the spike protein, the receptor-binding domain (RBD) or on virus-like particles; replication-incompetent vector vaccines; inactivated virus vaccines; live attenuated vaccines; inactivated virus vector vaccines that display the spike protein on their surface; replication-competent vector vaccines; DNA vaccines and RNA vaccines) are being used [24,25]. The details about the vaccines in development are available at https://www.who.int/publications/m/item/draft-landscape-of-covid-19-candidate-vaccines by the World Health Organization (WHO) [24]. After the emergency approval of two candidate vaccines based on mRNA technology, there is need to demonstrate the benefits of vaccination [26]. As we pass through 2020–2021 influenza season, there is still limited and inadequate data on symptoms that directly distinguish mild and moderate COVID-19 patients from ILI patients. Therefore, we retrospectively investigated the ILI outpatients for the presence of SARS-CoV-2.

## 2. Materials and Methods

### 2.1. Ethical Considerations

Ethics approval and consent to participate: All procedures performed in this study involving clinical specimen were in accordance with the ethical standards of the institutional and national research committee and with the 1964 Helsinki Declaration and its later amendments or comparable ethical standards. The study was approved by the Institutional Review Board at King Fahad Medical City (IRB Log No. 19-477 approved on 25 September 2019) for influenza vaccine effectiveness study, started from 7 November 2019. When the COVID-19 pandemic started, the collection of samples stopped on the 2nd of March 2020. To screen those samples for SARS-CoV-2, we applied for another IRB (Log No. 20-161 approved on 23 March 2020).

### 2.2. Study Specimens

The study specimens were obtained after written informed consent from a population (*n* = 739) attending the outpatient department (OPD) at our hospital. The handling of respiratory samples, as well as preparation of aliquots and viral RNA extraction, was performed using appropriate personal protective equipment in the biosafety level 3 laboratory. All samples testing positive for influenza virus and SARS-CoV-2 (*n* = 37) by real-time reverse transcription PCR (rRT-PCR) assay from 7 November 2019 to 2 March 2020 were used for this study (Table 1). The clinical datasets used and analyzed during the current study are available from the corresponding author on request.

### 2.3. Laboratory Investigations

Nasopharyngeal swab specimens were collected for laboratory-based investigations. A case of influenza like illness (ILI), according to our Ministry of Health definition, is fever >38 °C and cough with onset within the last ten days. Demographic information, medical history and outcome information were collected. Respiratory specimens were used for the detection of influenza viruses [27] and SARS-CoV-2 envelop (E) and ORF1ab genes [28] based rRT-PCR assays.

### 2.4. Real-Time Reverse Transcription PCR for Detection of Influenza and SARS-CoV-2

Viral RNAs were extracted using Qiagen viral RNA mini kit (Qiagen, Germantown, MD, USA) according to the manufacturers’ instructions. The detection of influenza viruses was performed by RT/q-PCR with Fast Track Diagnostic (FTD) Respiratory pathogens 21 plus kit (Biomerieux, Marcy-l’Étoile, France) following the manufacturer’s protocol. Briefly, 10 µL of the extracted nucleic acid was used as a template in each reaction. The thermal cycle amplification condition includes reverse transcription for 15 min at 42 °C, denaturation for 3 min at 94 °C followed by 40 cycles for 8 s at 94 °C, and 34 s at 60 °C. Real-Time reversed transcription PCR assay targeting E-gene and RdRp (ORF1ab) of SARS-CoV-2 was performed as previously described [28]. Briefly, all reactions were carried out in a 25 µL reaction volume; containing 5 µL of RNA, 12.5 µL of 2× reaction buffer provided with the Superscript III one step RT-PCR system with Platinum Taq Polymerase (Invitrogen, Darmstadt, Germany; containing 0.4 mM of each deoxyribont triphosphates (dNTP), 3.2 mM magnesium sulphate), 1 µL of reverse transcriptase/Taq mixture from the kit, 0.4 µL of a 50 mM magnesium sulphate solution (Invitrogen) and 1 μg of nonacetylated bovine serum albumin (Roche, Basel, Switzerland). Thermal cycling was performed initially at 55 °C for 10 min for reverse transcription, followed by 95 °C for 3 min and then 45 cycles of 95 °C for 15 s, 58 °C for 30. The limit of detection (LOD) was 10 RNA copies/mL.

### 2.5. Surveillance of Influenza in Saudi Arabia

Surveillance system for detection of ILI cases and the causative agent has been established since 2009 [29]. The total outpatient numbers along with the number of ILI cases were reported on a weekly basis by one of the sentinel hospitals, and clinical samples were also collected from ILI patients. In this study, we investigated the presence of SARS-CoV-2 among ILI patients visiting the outpatients with mild symptoms of fever >38 °C and a cough or sore throat. A total of 739 throat swabs collected from ILI patients in the 16-week period between November 2019 (week 44 of 2019) and March 2020 (week 9 of 2020) were re-examined.

### 2.6. Genome Amplification, Sequencing and Phylogenetic Analysis

We studied the evolutionary relationships of SARS-CoV-2 by reconstructing the phylogenies of the reference virus from Wuhan, China, and reported sequences until 2 March 2020. We performed complete genome sequencing of SARS-CoV-2 isolated from 17 outpatient’s visiting ILI patients. RNA was extracted from nasopharyngeal swabs and then reverse transcribed into cDNA using the High-Capacity cDNA Reverse Transcription Kit (Applied Biosystems, Baverly, MA, USA). Viral genomes were enriched from cDNA templates by PCR using a set of 58 pairs of overlapping primers designed to cover the entire SARS-CoV-2 genome. Amplified viral segments were then equally pooled and purified with the Qiaquick PCR purification kit (Qiagen, Germantown, MD, USA) before sequencing on the Illumina MiSeq instrument (Illumina, San Diego, CA, USA).

Paired-end sequencing libraries were prepared using the Nextera XT library preparation DNA kit (Illumina, San Diego, CA, USA) according to the manufacturer’s instructions. SARS-CoV-2 reads were filtered out by mapping to the viral reference sequence (GenBank accession: MN908947.3) using Bowtie2 version 2.3.4.1 [30] and SAMtools, version 1.8 [31]. Remaining reads were trimmed with Cutadapt, version 2.8 [32] to remove the sequences of the primers used for amplifications and then assembled with SPADES, version 3.13.0 [33]. The complete genome sequences from this study were compared to representatives of known SARS-CoV-2 clades available in the GISAID database (Global Initiative on Sharing All Influenza Data), including all those originating from our country (n = 112). Phylogenetic trees were constructed using the NextStrain nCoV automated pipeline using the assembled genomes and associated metadata as inputs (i.e., collection date and patient demographics and travel history). Assignment to the new dynamic nomenclature proposed by Rambaut et al. was determined using the Pangolin software (github.com/hCoV-2019/pangolin) [34]. A single-nucleotide polymorphisms (SNPs) distance in sequenced isolates was calculated from a MAFFT, version 7.455 [35] alignment of genome sequences after masking the first 130 and last 100 bases corresponding to 5′ and 3′ UTRs using an in-house python script. The complete genome sequences for 17 isolates were submitted to GenBank under accession numbers (MT755883-MT755899).

### 2.7. Statistical Analysis

The demographic and clinical characteristics were analyzed, and differences assessed for significance between groups by independent *t*-test. We used chi-square test of independence to examine the possible relation between influenza vaccination and clinical presentation in ILI patients positive for SARS-CoV-2. According to the case definition of ILI and COVID-19, we examined four clinical symptoms that ILI patients presented with fever, cough, sore throat and shortness of breath. Statistical analyses were performed using the IBM SPSS Statistics software (version 22.0, IBM Corporation, Armonk, NY, USA).

## 3. Results

We started collection of ILI nasopharyngeal samples (*n* = 739) on November 7, 2019 for an influenza vaccine effectiveness study, which was disrupted by the COVID-19 pandemic in March 2020. Since both ILI and COVID-19 case definitions overlap significantly, we therefore investigated the presence of SARS-CoV-2 in specimen belonging to ambulatory ILI patients visiting the outpatients with mild symptoms of fever >38 °C and a cough or sore throat. Nasopharyngeal samples collected from ILI patients in the 16-week period between 7 November 2019 (week 44 of 2019) and 1 March 2020 (week 9 of 2020) were re-examined. We re-investigated our ILI specimens for the presence of SARS-CoV-2. We found 37 samples positive for SARS-CoV-2 mostly from the month of February. We submitted those sequences to GenBank at the end of our experimental work in July with accession numbers (MT755883-MT755899).

### 3.1. Shortness of Breath in ILI Patients

Influenza vaccination was not statistically associated with SARS-CoV-2 infection (p = 0.60). The demographic and clinical data for the ILI patients in our study are described in Table 1. The study included 242 males and 497 females, with age ranging from 14 to 70 years (median age, 33 years). The male to female ratio was 1:2 and they were all adults (age range: 22–56 years). Out of 739 ILI samples collected for influenza vaccine effectiveness study from November 2019 to March 2020, we found 37 samples positive for SARS-CoV-2. The first positive case dated back to 30 January 2020 and the last case was 1 March 2020. During this time, influenza virus was still active but we could not find any influenza co-infection apart from three cases only (Table 2), which was not statistically significant in either group.

**Figure 1:**
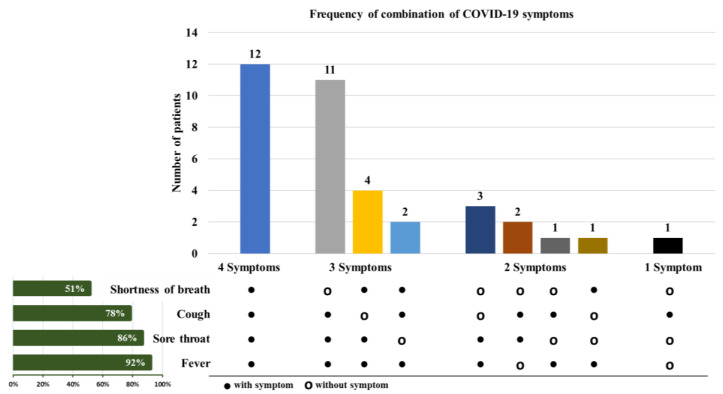
Distribution and combination of symptoms associated with 37 ILI cases positive for SARS-CoV-2. The variation in combination of symptoms observed in confirmed COVID-19 cases for the four most frequently observed symptoms (fever, sore throat, cough and shortness of breath) is represented by the colored vertical bars. The horizontal green bars on the left show the percentage of symptom occurrence in any combination with other symptoms. The black circles (•) highlight the symptoms and combination of symptoms experienced by COVID-19 patients. The open circles (ο) indicate the symptoms and combination of symptoms not experienced by the COVID-19 patients. Dark blue vertical bar represents number of patients with all four symptoms, gray, orange and light blue vertical bars indicate group with different combinations of three symptoms, Navy blue, dark brown, dark gray and brown bars indicate group with different combinations of two symptoms and black bar indicates group with one symptom only.

The case definition for ILI overlaps with the mild cases of COVID-19 due to presence of fever, sore throat, cough and shortness of breath. Therefore, we used chi-square test of independence to examine the association between influenza vaccination and clinical presentation in ILI patients positive for SARS-CoV-2. We examined four clinical symptoms that ILI patients presented with fever, cough, sore throat and shortness of breath (Figure 1). There was a significantly lower (*p* = 0.046) reported shortness of breath in SARS-CoV-2 patients with history of influenza vaccination than non-vaccinated SARS-CoV-2 patients presenting with ILI. However, there was no significant association between influenza vaccination and other clinical symptoms such as fever (*p* = 0.73), cough (*p* = 0.72) and sore throat (*p* = 0.69) respectively.

### 3.2. Surveillance of Influenza in Saudi Arabia

**Figure 2:**
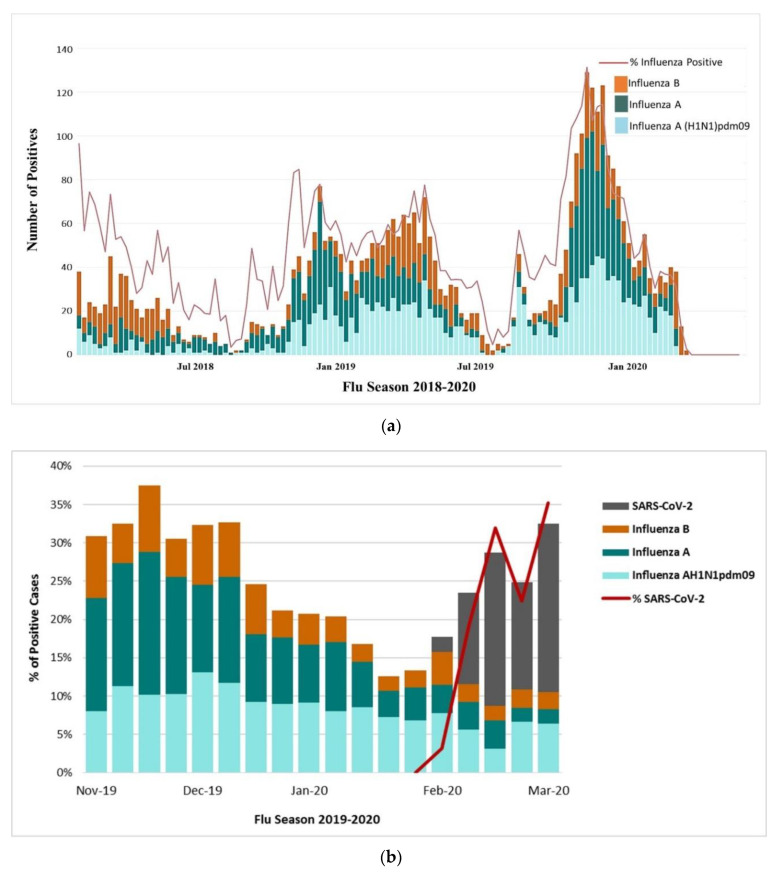
Distribution of specimens positive for influenza by subtype and % positive. (**a**) The positive specimens during three flu seasons 2018–2020 [36]. (**b**) The percentage of influenza and COVID-19 positive cases during 2019–2020 flu season.

The study time period overlapped with the winter peak of influenza and other severe acute respiratory illnesses or pneumonia (Figure 2a,b). There was an increase in the number of ILI cases in all age groups from the middle of November to the end of January 2020 (Figure S1). We observed a 5-fold increase in the 15–49 years’ group during the study period (week 44 of 2019-week 9 of 2020). The average percentage increase of ILI patients in all outpatients was 10.24% (Figure S1). The overall incidence of ILI cases during 2019–2020 influenza season was considerably higher than previous years (Figure 2a,b). It indicates the need to differentiate between influenza-related infections and suspected cases of SARS-CoV-2.

We noticed that SARS-CoV-2 positivity rate was small based on weekly sample size through the entire month of February which might indicate the slow spread of virus in the beginning of the outbreak. During the last week of February, the number of SARS-CoV-2 positive cases started to increase (Figure 2b). We also noticed that the SARS-CoV-2 positivity rate was small based on weekly sample size through the entire month of February which might indicate the slow spread of virus in the beginning of the outbreak. During the last week of February, the number of SARS-CoV-2 positive cases started to increase (Figure 2b).

### 3.3. Genetic Sequencing of SARS-CoV-2 Isolates

We selected 17 representative samples with the highest RNA copy number for full genome sequencing. We used temporal and spatial visualization to understand SARS-CoV-2 evolution through space and time that involved filtering large amounts of data by Nextstrain [37]. This also allowed us to perform simultaneous investigations of phylogenetic and spatial relationships, with GISAID clade based data expressed through colorings (Figure 2a,b).

**Figure 3:**
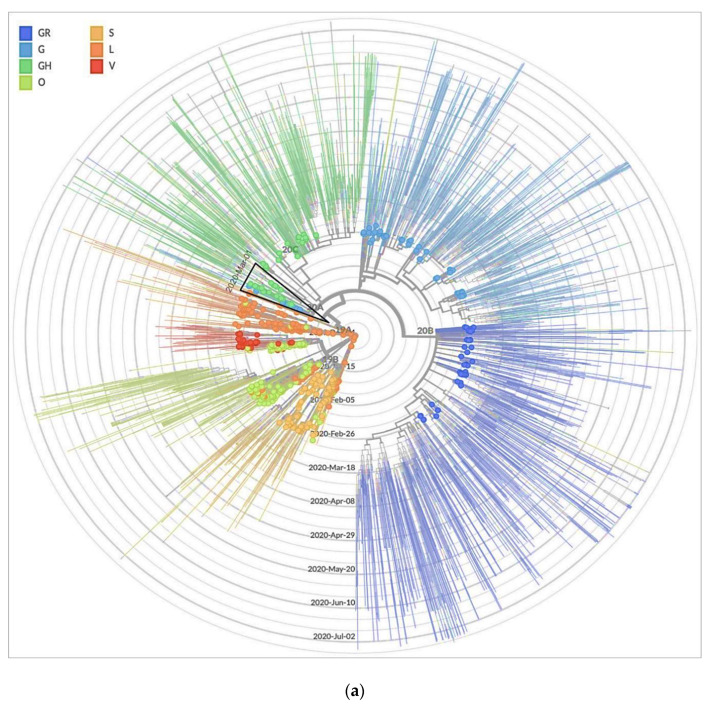

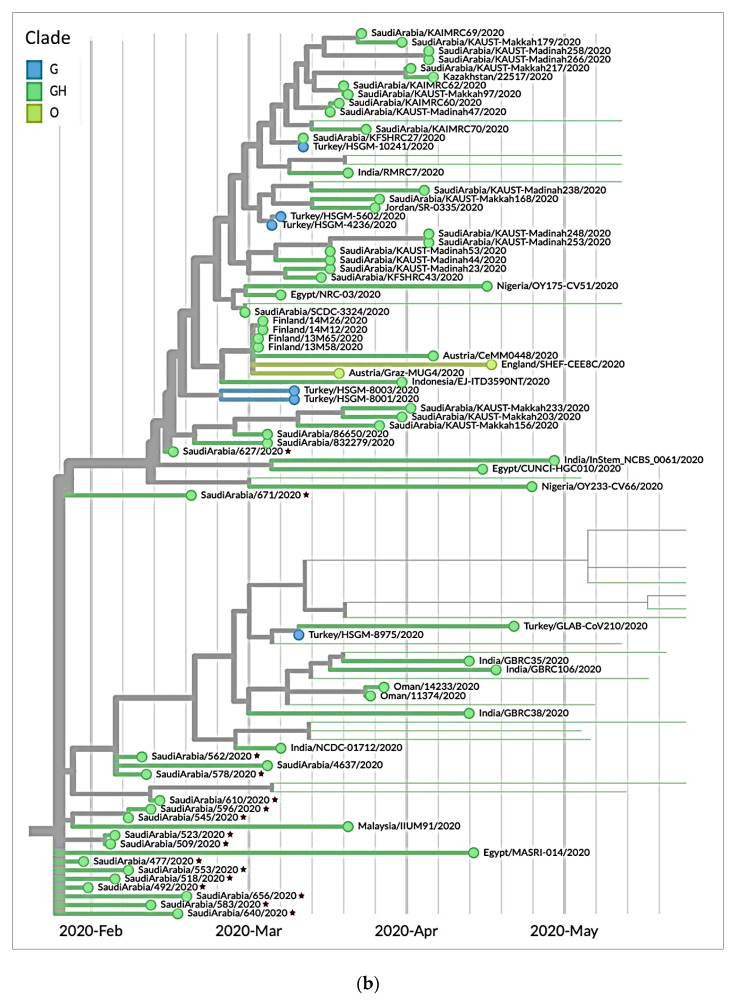
(**a**). Spatial and temporal distribution of complete SARS-CoV-2 genomes by radial Maximum Likelihood phylogeny reconstruction. Phylogenetic reconstruction of SARS-CoV-2 strains, with clades GR, G, GH, O, S, L and V, circulating globally between 20-12-2019 to 02-03-2020. Black triangle shows the clustering of 17 study sequences belonging to GH clade (dark green circles) until March 1. (**b**) Phylogenetic analysis of selected sequences for early introductions and circulation of SARS-CoV-2 belonging to GH clade (based on GISAID platform) represented by dark green circles. Black stars represent our study sequences dated from 3 February to 1 March 2020. The tips of the tree branches are shaped and colored according to the clade. Branch lengths are proportional to the number of nucleotide substitutions from the reference and tree root Wuhan/Hu-1/2019 (GenBank accession number MN908947). GISAID clades are indicated in the color legend in the left corner of the figure. Strain names of the sequences discussed in this study are indicated next to their corresponding tips in the tree.

We studied the evolutionary relationships of SARS-CoV-2 by reconstructing the phylogenies of the reference virus from Wuhan, China, and reported sequences until 2 March 2020. The phylogenetic tree (Figure 3a) was subdivided into seven clades corresponding to the seven SARS-CoV-2 strain types GR (dark blue), G (light blue), GH (dark green), S (brown), V (red), L (orange) and O (others; light green). The four strain types S, G, GR and GH are scattered all over the world (Figure 3a). According to GISAID (update of the 21st of July 2020), G clade first appearance in February 2020, which diverged to the GH and GR clades, while the S strain was mainly found mainly in Europe. Most of the study viruses (*n* = 14) showed close relationship among each other and clustered in the GH clade, which is characterized by two amino acid changes in spike gene (D614G) and NS3 (Q57H) (Figure 3b). Most of our study viruses showed amino acid changes in the spike and different non-structural proteins. This sub-group caused silent community spread in the western part of Saudi Arabia as well as to India, Jordan and Australia (Figure 3b). The second sub-group of our study viruses (*n* = 3) also belonged to GH clade but clustered separately from the first sub-group, resulting in spread to various countries (Australia, Turkey, Egypt, Bangladesh and India).

### 3.4. Structural Predictions of the Proteins

For the analysis of sequence variations in the spike, RNA-dependent RNA polymerase (RdRp) and NS3 proteins among sequenced SARS-CoV-2 genomes, the 3D structures were generated with initial models of the respective proteins using the fully automated protein structure homology-modelling server SWISS-MODEL, accessible via the ExPASy web server and protein structure figures were produced in PyMOL [38].

**Figure 4:**
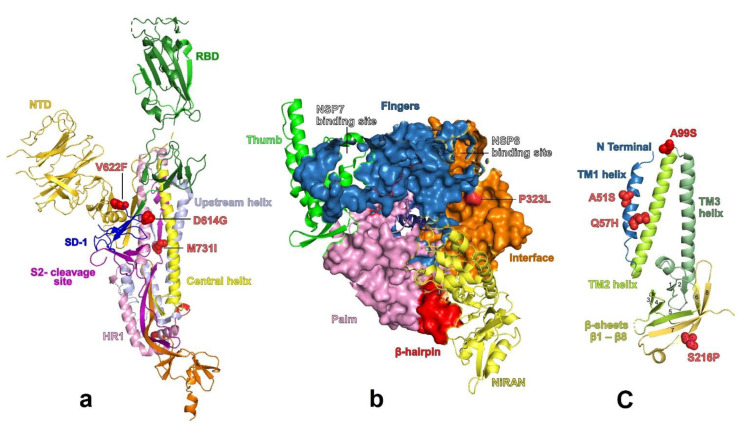
Homology structures of spike promoter, RdRp (polymerase) and Orf3a (NS3), with structural mapping of amino acid changes. (**a**) Cartoon representation of the S. promoter for detailed architecture with amino acid changes (red spheres) based on template (pdb id: 6VSB). RBD: receptor-binding domain; SD-1: subdomain-1; S2: subunit 2 of the spike glycoprotein; HR: heptad repeat-1 region. (**b**) Surface structure of nsp12 (polymerase) colored by domains (pdb id: 6XEZ). The structure of nsp12 is defined by thumb (green), followed by the finger subdomain (blue), connecting to NiRAN domain (yellow) and the palm subdomain (pink) at amino (N) terminal binding at the back side to the C-terminal RdRp. The interface domain (brown) connects the NiRAN domain to the finger subdomain. NSP: nonstructural protein; NiRAN: nidovirus RdRp-associated nucleotidyltransferase. (**c**) Structure of Orf3 (NS3) in cartoon representation with trans-membrane region (3 helices) in protomer with amino acid changes shown as red spheres (pdb id: 6XDC). TM: transmembrane.

Comparison of the SARS-CoV-2 genomes from our study identified two of the previously reported D614G, V622F and one novel M731I substitutions in the spike protein (Supplementary Materials Table S1, Figure 4a). All genomes had the D614G substitution, whereas the remaining variations were identified each in a single genome. Structural mapping of these modifications showed that the dominant variation at residue 614 is located on the surface of S protomer and forms a connection with the protomer of a neighboring chain (Figure 4a). The amino acid change at position 622 was also seen in the same promoter, whereas 731 was located in S2 domain primarily affecting the disulphide bonding (Figure 4a).

Nsp12 forms the core catalytic subunit bound with a nsp7-nsp8 heterodimer to complete the polymerase complex of SARS-CoV-2. The structure of nsp12 is defined by nidovirus RdRp-associated nucleotidyltransferase (NiRAN) domain and the palm subdomain at amino (N) terminal (Figure 4b). It binds at the back side to the C-terminal RdRp. The interface domain connects the NiRAN domain to the finger subdomain. All of our study sequences had both P323L (nsp12) and D614G (Spike) substitutions. Therefore, we constructed the structural homology models to further analyze the coevolution of P323L and D614G (Figure 4a,b). The most prevalent substitution found in orf3a (NS3) was Q57H followed by A51S and S216P (Figure 4c). Q57 is responsible for the formation of hydrophilic constrictions in the transmembrane helix 1 (TM-1).

## 4. Discussion

We investigated the presence of SARS-CoV-2 in the local ambulatory patients presenting with ILI in order to understand the role of ILI cases in the current pandemic especially cases with mild illness. We found 37 COVID-19 positive cases during the investigation of ILI cases collected during last influenza season 2019–2020. Although 68% of our COVID-19 cases had history of vaccination, the influenza vaccination had no direct effect on COVID-19 positivity. The SARS-CoV-2 genomes in this study provide more insight into the clades distribution and variants circulating in Saudi Arabia at the beginning of the outbreak and later during the pandemic. The amino acid substitutions observed among structural proteins will pave the way for future functional studies.

Seasonal influenza and coronavirus disease 2019 (COVID-19) have overlapped this year (Figure 1a), burdening the health care systems. In 2018–2019, Saudi Arabia witnessed less ILI cases caused by influenza (Figure 1a). Transmission of SARS-CoV-2 from asymptomatic or pre-symptomatic persons is increasingly evident and is estimated to be 20–40% of all COVID-infected subjects [39,40,41]. Both symptomatic and asymptomatic patients of COVID-19 have been reported to show similar viral loads, which suggests the transmission potential of asymptomatic or minimally symptomatic patients [16,42]. Our findings are in line with reports that transmission may occur early in the course of SARS-CoV-2 infection. Community transmission of SARS-CoV-2 may occur from symptomatic, asymptomatic or mildly symptomatic people carrying the virus. On Diamond Princes cruise ship, 712 persons were infected with SARS-CoV-2 among the 3711 passengers and crew members, 410 (58%) of whom were asymptomatic or pre-symptomatic at the time of testing. However, true asymptomatic SARS-CoV-2 infection were confirmed in only 90 persons (12.6%) [43]. Identification of asymptomatic or mildly symptomatic persons is critical in curbing silent community spread and would enhance public health strategies to contain COVID-19.

Estimation of the prevalence and contagiousness of undocumented COVID-19 infected people is critical for understanding the overall prevalence of this disease. Seroprevalence studies have reported the estimated rate of transmission of undocumented infections per person at >4% in the US [44], 5% in Spain [45], 3.8% in China [46] and 5% in Kenya [47]. Pharyngeal shedding of virus reaches high levels in mild symptomatic patients of COVID-19 during the first week of symptoms with a peak >7 × 10^8^ RNA copies per mL on the fourth day [12]. We found 37 (5.25%) clinical specimen positive for SARS-CoV-2 among the 739 tested patients with mild ILI during the period of 1 November 2019 until 2 March 2020. Similar proportions (5.3%) of mild ILI cases caused by SARS-CoV-2 have been reported from the USA (7/131 patients) [48]. However, community spread of SARS-CoV-2 in Wuhan from early January was suggested on the basis of detection in 9 out of 640 patients with influenza-like illness (1.4%) between October 2019 to January 2020 [21].

The similarity of clinical manifestations and case definition between the influenza and coronaviruses make the differentiation very difficult [49,50]. The Saudi Ministry of Health COVID-19 case definition of suspected COVID-19 is a person with acute respiratory illness and in the 14 days prior to symptoms onset, met at least history of travel abroad or identified high risk area; a close contact prior to symptoms onset to a confirmed COVID-19 case; or working in or attended a healthcare facility where patients with confirmed COVID-19 were admitted. Thus, testing for SARS-CoV-2 was not indicated for persons with ILI in the absence of epidemiological link to confirm COVID-19 cases. Most surveillance systems including MERS-CoV only capture cases that are severe enough to cause a person to seek medical care, yet a potentially large number of mild cases remain undetected, called “tip of iceberg” [51]. Additionally, the overall number of patients positive for influenza and other respiratory viruses during the COVID-19 period have decreased significantly when compared with that in the same period of the last two years, reflecting that public health interventions can effectively control the spread of common respiratory viruses [52].

The phylogenetic data presentation helped us understand the geographical spread of the SARS-CoV-2 alongside the underlying genomic data that supports this geographic reconstruction. Maximum likelihood ancestral state reconstruction of discrete traits such as country of isolation allowed identification of probable transmission events in the study sampled data, together with inferred probability distributions of ancestral state at each node. SARS-CoV-2 evolution into different lineages shown by varied levels of virulence and transmissibility could be possible. However, currently there is no evidence of evolution of distinct phenotypes in SARS-CoV-2 based on virulence and transmission [30,53]. G clade sequences appeared first in January, diverging into GH and GR clades in February. All of our sequences clustered in the GH clade. Our samples nested with those from Europe in March; however, Nextstrain analysis shows the presence of GH clade sequences during the month of February reported from Senegal, Canada, Belgium, Singapore, Lebanon and France [54,55]. SARS-CoV-2 might have been circulating elsewhere in the world but there was little PCR testing during February for COVID-19, which might have also affected availability of sequencing data. Maybe that is why, to our advantage during February, few sequences were reported originating from COVID-19 cases and nothing from ILI. We believe our findings will greatly add value to the current understanding and divergence of GH clade sequences.

Structural mapping of these modifications showed that the dominant variation at residue 614 is located on the surface of S protomer and forms a connection with the protomer of a neighboring chain (Figure 4a). The amino acid change at position 622 was also seen in the same promoter, whereas 731 was located in S2 domain, primarily affecting the disulphide bonding (Figure 4a). Substitution or deletion of residue 731 from its C-terminal is known to be associated with gradual down regulation of cyclooxygenase-2 (COX-2) promoter [56]. The role of 614 amino acid change in the S1–S2 junction for increased transmission and less infectivity is already established [57,58]. However, the results of the temporal analysis of the mutation frequency of P323L (nsp12), and D614G (S-protein) show that P323L was consistently present in the viruses and started co-evolving with D614G sometime late January 2020 [59]. Methionine to isoleucine (Spike, M731I) M to I substitution in HIV-1 reverse transcriptase provides resistance to nucleoside analog 2′,3′-dideoxy-3′thiacytidine [60]. Interestingly, there was a non-synonymous substitution (Proline to leucine; P323L) in RNA Dependent RNA Polymerase (RdRp) region next to the nsp8 binding site in majority of Saudi SARS-CoV-2 sequences. Our future work will be based on the validation of the effect of this substitution on RdRp and any possible resistance against certain antiviral or inhibitors such as bisheteroarylpiperazines (BHAPs) like in the case of HIV-1 reverse transcriptase [61]. Whether the functional competence of polymerase is affected due to these amino acid changes remains to be seen. Although there has been talk about the role of D614G mutation and enhancement of infectivity, it is difficult to associate it to a single mutation. The co-existence of D614G (spike) and P323L (nsp12) might contribute to replication and infectivity along with host factors.

The most prevalent substitution found in orf3a (NS3) was Q57H, followed by A51S and S216P (Figure 4c). Q57 is responsible for the formation of hydrophilic constrictions in the transmembrane helix 1 (TM-1). However, the substitution at this residue with histidine has been shown to have no changes in functional dynamics of membrane channel and evolutionary origins of SARS-CoV-2 [37,62]. For the remaining two substitutions, the change or influence in oligomerization or membrane channel function (intra- and extracellular) remains unknown.

The limitation of our study was that we could not explain the possible role of these viruses in the silent transmission and community spread. Identification of transmission chains and clusters depends on the extent of presymptomatic and asymptomatic transmission. We could not evaluate the contribution of presymptomatic and asymptomatic transmission based on recent individual-level data regarding infectiousness. We found that the majority of incidences in our study may be attributed to silent transmission from a combination of the mild infections originating from ILI. For future studies, our findings show that mild symptomatic infections must be supplemented by rapid contact tracing and testing that identifies asymptomatic and presymptomatic cases to reduce the risk of resurgence.

## 5. Conclusions

In conclusion, assessment of the community transmission among patients with mild COVID-19 illness, particularly those without epidemiological link for acquiring the virus, is of utmost importance to guide policy makers to optimize public health interventions. The detection of SARS-CoV-2 in ILI cases shows the importance of and ILI surveillance systems and warrants its further strengthening to mitigate the ongoing transmission of SARS-CoV-2. It also indicates the need to differentiate between influenza-related infections and suspected cases of SARS-CoV-2. Functional validation of structural changes observed in various proteins of SARS-CoV-2 requires further studies.

## Figures and Tables

**Table 1:** Demographics and clinical characteristics.

Baseline Variables	All Patients	SARS-CoV-2 Pos	SARS-CoV-2 Neg	*p*-Value
	*n* = 739	*n* = 37 (5%)	*n* = 702 (95%)	
Characteristics				
Median Age	33.4 ± 8.5	33.9 ± 9.6	33.4 ± 8.5	
Gender				
Men	242 (33%)	8 (3.3%)	234 (96.6%)	*p* = 0.13 *
Women	497 (67%)	29 (5.8%)	468 (94.1%)	
Flu Vaccination				
Vaccinated	470 (64%)	25 (68%)	445 (63%)	*p* = 0.60 *
Non-vaccinated	269 (36%)	12 (32%)	257 (67%)	

* Not significant at *p* < 0.05.

**Table 2:** Underlying clinical symptoms in COVID-19 patients.

	SARS-CoV-2 Positives (*n* = 37)				
	Vaccinated		Non-Vaccinated		
Symptoms	With	Without	With	Without	
Fever	22 (59%)	3 (8%)	11 (30%)	1 (3%)	*p* = 0.73
Sore throat	22 (59%)	3 (8%)	10 (27%)	2 (6%)	*p* = 0.69
Cough	20 (54%)	5 (14%)	9 (24%)	3 (8%)	*p* = 0.72
Shortness of breath	10 (27%)	15 (41%)	9 (24%)	3 (8%)	*p* = 0.04 *
Co-infection	7 (19%)	18 (49%)	3 (8%)	9 (24%)	*p* = 0.84

* Number of patients showing shortness of breath with a history of flu vaccination were significantly (*p* > 0.05) lower than non-vaccinated patients.

## Data Availability

Data is contained within the article and supplementary material.

## Supplementary Materials

The following are available online at https://www.mdpi.com/1999-4915/13/1/136/s1, Figure S1: The number of ILI cases in all age groups from the middle of January 2018 to March 2020. Table S1: Comparison of complete genome sequence variants for the SARS-CoV-2 isolated from the patients in Saudi Arabia, 2020, and reference strain.
