# Supplementary material for: Structural Mapping of Mutations in Spike, RdRp and Orf3a Genes of SARS-CoV-2 in Influenza Like Illness (ILI) Patients
Source: Viruses. 2021 Jan 19;13(1):136. doi: 10.3390/v13010136 (PMC7835825; doi:10.3390/v13010136)
Supplement: Supplementary file 1 [file viruses-13-00136-s001.pdf]

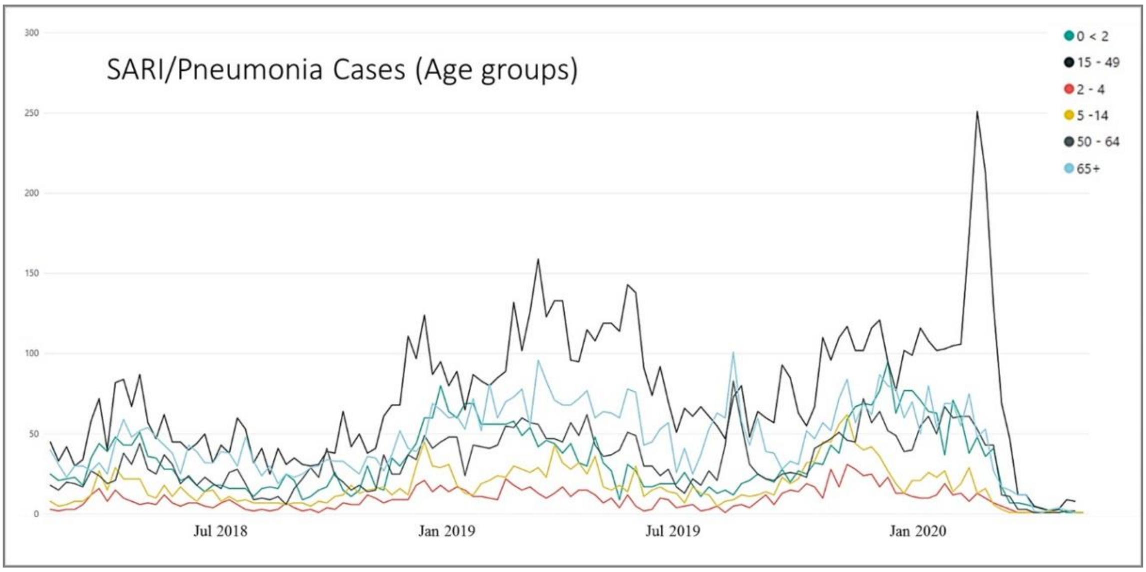

Figure S1. The number of ILI cases in all age groups from the middle of January 2018 to March 2020. We observed a 5-fold increase in the 15–49 years' group during the study period (week 44 of 2019—week 9 of 2020). The average percentage increase of ILI patients in all outpatients was 10.24%.

Table S1. Comparison of complete genome sequence variants for the SARS-CoV-2 isolated from the patients in Saudi Arabia, 2020, and reference strain.

SARS-CoV-2 strains	NSP1	NSP2	NSP3	NSP4	NSP9	NSP12	SPIKE	NS3	N
**MN735679-Saudi Arabia-477-2020**	-	S138L	K1771R	-	-	P323L, Y606C	D614G	Q57H	-
**MN735681-Saudi Arabia-492-2020**	-	-	D853G	-	-	P323L	D614G, V622F	Q57H	-
**MN735682-Saudi Arabia-509-2020**	-	-	E1450K	-	-	P323L	D614G	Q57H	-
**MK858156-Saudi Arabia-518-2020**	-	-	-	-	-	P323L	D614G	Q57H, A51S	-
**MK858157-Saudi Arabia-523-2020**	-	-	E1450K	-	K36N	P323L	D614G	Q57H	-
**MK858158-Saudi Arabia-545-2020**	-	-	-	-	-	P323L	D614G	Q57H	-
**MK858159-Saudi Arabia-553-2020**	G150C	A31V	M1556T, T1590A	-	-	P323L	D614G	Q57H	-
**MK858160-Saudi Arabia-562-2020**	-	-	-	-	-	P323L	D614G	Q57H	-
**MK858161-Saudi Arabia-578-2020**	-	-	-	-	-	P323L	D614G	Q57H	S194L, P383L
**MK858162-Saudi Arabia-583-2020**	-	-	-	E183G	-	P323L	D614G	Q57H	P326L
**MK858163-Saudi Arabia-596-2020**	-	-	-	-	-	P323L	D614G	Q57H	-
**MK858164-Saudi Arabia-610-2020**	-	-	-	-	-	P323L	D614G	Q57H	S194L

**KT806006-Saudi Arabia-627-2020**	-	-	-	-	-	P323L	D614G	Q57H	-
**KT805971-Saudi Arabia-640-2020**	-	-	-	-	-	P323L	D614G	Q57H	-
**MG011342-Saudi Arabia-656-2020**	-	-	V253I	-	-	P323L	D614G	Q57H, A99S	-
**MG912606-Saudi Arabia-671-2020**	-	-	A994V	-	-	P323L	D614G	Q57H	-
**MG912606-Saudi Arabia-677-2020**	-	-	-	-	-	P323L	D614G, M731I	Q57H, S216P	-
